# GASTRICHIP: D2 resection and hyperthermic intraperitoneal chemotherapy in locally advanced gastric carcinoma: a randomized and multicenter phase III study

**DOI:** 10.1186/1471-2407-14-183

**Published:** 2014-03-14

**Authors:** Olivier Glehen, Guillaume Passot, Laurent Villeneuve, Delphine Vaudoyer, Sylvie Bin-Dorel, Gilles Boschetti, Eric Piaton, Alfredo Garofalo

**Affiliations:** 1 Hospices Civils de Lyon, Centre Hospitalier Lyon-Sud, Service de Chirurgie Viscérale et Endocrinienne, Pierre-Bénite Cedex 69495, France; 2 Université Lyon 1, EMR 3738, Oullins 69921, France; 3 Hospices Civils de Lyon, Unité de Recherche Clinique, Pôle Information Médicale Evaluation Recherche, Hospices Civils de Lyon, Lyon 69003, France; 4 Université de Lyon, EAM Santé Individu Société 4128, Lyon 69003, France; 5 Université Lyon 1, Lyon 69003, France; 6 Hospices Civils de Lyon, Centre Hospitalier Lyon-Sud, Service de Gastroentérologie, Pierre-Bénite Cedex 69495, France; 7 Hospices Civils de Lyon, Centre de pathologie Est, Bron 69577, France; 8 Reparto di Chirurgia Oncologica, Institutio Nazionale Tumori Regina, Roma 00144, Italia

**Keywords:** Gastric adenocarcinoma, Hyperthermic intraperitoneal chemotherapy, Oxaliplatin, Peritoneal carcinomatosis

## Abstract

**Background:**

In Europe, gastric cancer remains diagnosed at advanced stage (serosal and/or lymph node involvement). Despite curative management combining perioperative systemic chemotherapy and gastrectomy with D1-D2 lymph node dissection, 5-year survival rates of T3 and/or N + patients remain under 30%. More than 50% of recurrences are peritoneal and/or locoregional. The use of adjuvant hyperthermic intraperitoneal chemotherapy that eliminates free cancer cells that can be released into peritoneal cavity during the gastrectomy and prevents peritoneal carcinomatosis recurrences, was extensively evaluated by several randomized trials conducted in Asia. Two meta-analysis reported that adjuvant hyperthermic intraperitoneal chemotherapy significantly reduces the peritoneal recurrences and significantly improves the overall survival. As it was previously done for the evaluation of the extension of lymph node dissection, it seems very important to validate on European or caucasian patients the results observed in trials performed in Asia.

**Methods/design:**

GASTRICHIP is a prospective, open, randomized multicenter phase III clinical study with two arms that aims to evaluate the effects of hyperthermic intraperitoneal chemotherapy with oxaliplatin on patients with gastric cancer involving the serosa and/or lymph node involvement and/or with positive cytology at peritoneal washing, treated with perioperative systemic chemotherapy and D1-D2 curative gastrectomy. Peroperatively, at the end of curative surgery, patients will be randomized after preoperatively written consent has been given for participation. Primary endpoint will be overall survival from the date of surgery to the date of death or to the end of follow-up (5 years). Secondary endpoint will be 3- and 5-year recurrence-free survival, site of recurrence, morbidity, and quality of life. An ancillary study will compare the incidence of positive peritoneal cytology pre- and post-gastrectomy in two arms of the study, and assess its impact on 5-year survival. The number of patients to be randomized was calculated to be 306.

**Trial registration:**

EudraCT number: 2012-005748-12, ClinicalTrials.gov identifier: NCT01882933.

## Background

Stomach is the fourth most common digestive cancer [1], the second main cause of death from cancer in the world [2], and the fifth most common cancer in Europe [3].

### Surgery

Surgery remains the curative treatment of choice for stomach cancer. It consists of a radical subtotal or total gastrectomy with D1 or D2 lymph node Dissection. A logical and reasonable alternative (expert consensus) is therefore to carry out D1 dissection associated with pedicle dissection (common hepatic artery, left gastric artery and proximal splenic artery). This extension corresponds to D2 dissection for antral cancers. When this type of dissection without splenectomy or pancreactectomy is carried out for a cancer of the body or the upper-third of the stomach it is commonly termed D1.5 dissection. Extending the lymph node dissection remains controversial. Two randomized studies and a meta-analysis have shown no benefit from D2 dissection, although a notable benefit was observed in a sub-group of patients with lymph node metastasis [4-6]. More recently, two well-designed, single-arm studies [7,8] showed that a modified form of D2 dissection or D1.5 dissection, without splenectomy or pancreatectomy (which increases post-operative morbidity and mortality) has better results in terms of survival than D1 dissection, with acceptable levels of morbidity and mortality.

### Neoadjuvant, adjuvant and perioperative treatments

Systemic perioperative chemotherapy is recommended for the curative treatment of stomach cancer in Europe since the publication of the MAGIC [9] and Fédération Française des Centres de Lutte Contre le Cancer (FNCLCC) - Fédération Française de Chirurgie Digestive (FFCD) [10] trials. These studies included 503 and 224 patients respectively, presenting with a gastric adenocarcinoma or adenocarcinoma of the cardia treated with surgery associated, or not, with two or three preoperative, and three postoperative cycles of systemic chemotherapy (ECF or 5-Fluorouracil (5-FU) cisplatin). The 5-year survival rate was 36% and 38% respectively in the experimental arm, compared to 23% and 24% respectively in the control arm (surgery alone). The postoperative chemotherapy was not always feasible: only 40% of patients followed the postoperative treatment regimen in the MAGIC trial and 50% in the FNCLCC-FFCD trial.

Adjuvant chemo-radiotherapy was shown to be effective in a phase III study by MacDonald et al. [11] This tested chemotherapy (FUFA: 5-FU/Folinic Acid) prior to, and following chemo-radiotherapy (FUFA + 45 Gy) and demonstrated its efficacy in terms of median survival (36 *vs* 27 months). Two-thirds of the patients include were at stages T3 or T4 and 85% had node positive. The main criticism of this trial is that in 54% of cases lymph node dissection was D0. For many experts, this therefore limits the applicability of adjuvant chemo-radiotherapy following resection. This chemo-radiotherapy can be considered as an alternative for certain patients in good general and nutritional health with lymph node invasion having undergone adequate lymph node Dissection. A retrospective study suggested that replacing FUFA with simplified LV5FU2 (Leucovorin/5-FU) reduces toxicity [12]. Another non-randomized comparative study assessed postoperative chemo-radiotherapy for patients having undergone D2 dissection of over 85%, and reported a benefit from this adjuvant treatment for stages IIIA, IIIB and IV [13].

A recent meta-analysis of 17 randomized trials including a total of 3,838 patients reported a benefit in terms of overall survival from using systemic postoperative chemotherapy 5-FU *vs* surgery alone [HR 0.83 (95% CI 0.74-0.94)] [14].

### Peritoneal recurrence

Stomach cancer has the highest rate of peritoneal recurrence of all digestive cancers. After curative surgery, the main reason for treatment failure is peritoneal recurrence which, according to the literature, occurs in 40 to 60% of cases, despite extensive surgery including D2 lymph node dissection [15,16].

Several factors favorable to peritoneal recurrence have been identified: invasion of the serosa (T3, T4 tumors) [17,18], detection of free cancer cells in the peritoneal wash liquid [19,20], invasion of the lymph nodes [21], and signet ring cells adenocarcinoma [22].

### Rationale for hyperthermic intraperitoneal chemotherapy (HIPEC)

The failure rate for curative surgical treatment for patients with stomach cancer is mainly due to peritoneal recurrence. It would therefore seem appropriate to offer preventive treatment to reduce the risk of peritoneal recurrence in at-risk patients and thereby reduce the failure rate. The HIPEC technique is increasingly used in the curative treatment of primary and digestive peritoneal carcinomatosis, in association with cytoreductive surgery [23-25]. It is recommended for treating pseudomyxoma peritonei and peritoneal mesothelioma [25,26] and it is currently being assessed in France for use in the curative and preventive treatment of colorectal and ovarian carcinomatosis in several phase III studies funded by the French clinical research projects funding program (PHRC): PRODIGE 7/ACCORD 15/0608 (ClinicalTrials.gov number NCT00769405, ProphyloCHIP (ClinicalTrials.gov number: NCT01226394), CHIPOR (ClinicalTrials.gov number NCT01376752).

Intraperitoneal chemotherapy has the advantage of putting the intraperitoneal tumor tissue (which at the beginning of growth is small or non-vascularized) and free cancer cells into direct contact with high concentrations of cytotoxic agents, limiting the systemic concentrations and thereby the risk of toxicity. The cytotoxic effect of heating to 42.5°C has been demonstrated *in vitro*[27] and it has also been demonstrated that the hyperthermia increases the effectiveness of certain molecules (mitomycin C, cisplatin, oxaliplatin), either by increasing their cytotoxicity, or by increasing their penetration into the tumor tissue [28,29].

### Results for HIPEC used as a curative and preventive treatment for gastric cancer

For gastric carcinomatosis, the association of complete cytoreductive surgery (CCS) and HIPEC is the only therapeutic strategy to achieve long-term survival. A retrospective study of 159 patients with gastric carcinomatosis treated with CCS and HIPEC, reported a 5-year survival rate of 23%. These extended survival rates was obtained in rigorously selected patients presenting with local, resectable carcinomatosis [30]. A recent randomized phase III study demonstrated the benefit of HIPEC (cisplatin and mitomycin C) associated with CCS. Median survival was 11 months in the CCS + HIPEC group as compared to 6.5 months in the group receiving CCS alone (p = 0.046) [31]. The benefit of HIPEC was greater in patients with synchronous carcinomatosis.

Several asian authors have reported a potential benefit from using intraperitoneal chemotherapy with or without hyperthermia, as a complement to curative surgery, in the absence of carcinomatosis [32-34].

Fujimoto et al. [35] recruited 141 patients and showed that HIPEC significantly reduced the incidence of peritoneal recurrence (p < 0.001), and increased the survival rate (p = 0.03) with no postoperative adverse events. Yonemura et al. [36] randomized 139 patients into 3 arms: surgery alone, surgery with HIPEC and intraperitoneal chemotherapy without hyperthermia. The 5-year survival rate was 61% in the HIPEC group as opposed to 43% and 42% in the other two groups. In 2001 Kim and Bae [37] published the results of a controlled study on 103 patients presenting with a gastric carcinoma with invasion of the serosa, who underwent surgical resection alone or associated with HIPEC. The 5-year survival rate was significantly higher in the experimental group when stage IV patients were excluded (p = 0.0379). The most common types of recurrence were locoregional in the HIPEC group and peritoneal in the control group. Yan et al. [38] published a meta-analysis which also demonstrated that using HIPEC as an adjuvant treatment significantly improved the survival rates of patients with stomach cancer (HR = 0.60; CI 95% = 0.43 to 0.83; p = 0.002). This meta-analysis suggested that intraperitoneal chemotherapy delivered intraoperatively with hyperthermia was a more effective approach than the delayed regimen. More recently, another meta-analysis also showed the potential benefit of using HIPEC for patients with an advanced gastric cancer in an adjuvant setting [39]. The benefit of using HIPEC as an adjuvant treatment for advanced gastric cancer has been reported in several randomized studies and a meta-analysis [38]. However these studies included patients who were almost exclusively of Asian origin. It has been formally demonstrated that Asian and Caucasian gastric cancers differ in terms of epidemiology, diagnosis, treatment and prognosis. On the subject of lymph node dissection for example, several randomized studies in Asia and Japan have validated D2 dissection. In Europe, two randomized studies were carried out to assess the benefit of D2 dissection and the conclusions of these studies differ from the Asian studies [8,40].

Given that curative treatment failure in Western countries is mainly due to peritoneal recurrence and that a meta-analysis composed almost entirely of Asian studies suggests the benefit of HIPEC as an adjuvant treatment, a European study on a Caucasian population would seem to be warranted.

## Methods/design

### Protocol overview

**Figure 1:**
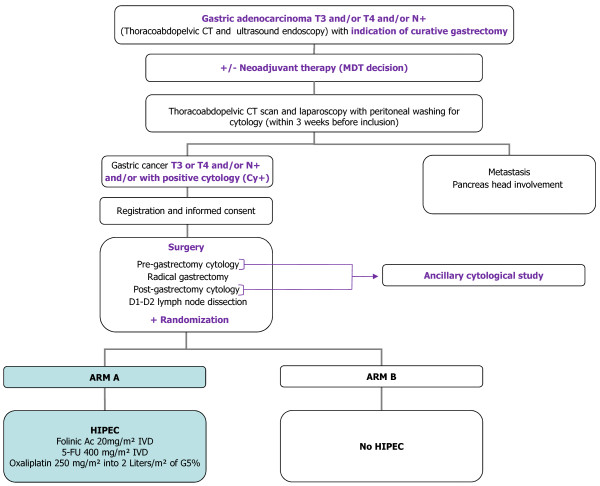
GASTRICHIP study flow-chart.

GASTRICHIP is a prospective, open, randomized multicenter phase III clinical study aimed to evaluate the effects of HIPEC with oxaliplatin on patients with gastric cancer involving the serosa and/or lymph node involvement and/or with positive cytology at peritoneal washing, treated by D1-D2 curative gastrectomy (Figure 1 – Flow chart study). Patients will be randomly assigned in a 1:1 ratio to: Arm A: curative gastrectomy with D1-D2 lymph node dissection + HIPEC with oxaliplatin versus Arm B: curative gastrectomy with D1-D2 lymph node dissection without HIPEC.

### Measures of outcomes and assessments

#### Primary outcome

Overall survival will be measured from the date of surgery to the date of death or to the end of follow-up (5 years).

### Secondary outcomes

Efficacy (3-year and 5-year recurrence-free survivals) and localization of recurrence, morbidity, and quality of life.

### Main inclusion criteria

Patients 18 < age ≤ 75 years old with Karnofsky index ≥ 70% with histologically evidenced resectable T3 or T4 gastric adenocarcinoma for which a curative gastrectomy is scheduled, with invasion into the serosa and/or lymph node metastasis (determined from data obtained by endoscopic ultrasound and chest, abdomen and pelvis Computed Tomography (CT) scan) and/or positive peritoneal cytology (sampled during the preoperative laparoscopy) and/or perforated gastric adenocarcinoma and/or Siewert III adenocarcinoma of the cardia for which a gastrectomy by exclusive abdominal laparotomy is scheduled [41].

### Exclusion criteria

The following criteria will exclude patients: prior malignant tumors with detectable signs of recurrence, gastric stump adenocarcinoma, presence of comorbidities, notably serious chronic diseases or organ failure, pregnancy or breastfeeding, contraindication to any drug contained in the chemotherapy regimen, life threatening toxicity before surgery, distant metastases (liver, lung. ovaries, etc.), tumoral infiltration of the head or body of the pancreas, patients presenting an adenocarcinoma of the cardia Siewert I or II, existence of any macroscopic peritoneal implants, patients with clinically significant ascites (> 500 cc) even if cytology is negative for cancer cells, in the absence of other non-malignant causes of ascites.

### Randomization

At the time of gastrectomy, if the patient has given informed, written consent and meets inclusion criteria, he will be peroperatively randomized, using an interactive Web response system. Randomization will be balanced and stratified by investigating center; because of the center’s influence in the most studies that evaluated HIPEC, and by the presence or absence of signet ring cells, based on the findings of the preoperative biopsies because of the recent demonstration of its particular prognosis and its relative chemoresistance.

### Treatments

#### Perioperative treatment

All validated perioperative treatments of gastric cancer will be authorized. For patients receiving neoadjuvant chemotherapy, surgery will be scheduled 2 to 4 weeks after the last course of systemic chemotherapy.

The pre- and the post-surgery chemotherapy regimen administrated to a patient must be the same except in case of toxicity, or progression.

### Pre-therapeutic work-up

Patients eligible for the study will be seen in clinics following the explorative laparoscopy to check the inclusion and exclusion criteria. The patient will be required to give written informed consent to participate to this clinical study before any non routine screening tests or evaluations are conducted. Patients eligible for the study will be seen in clinics following the explorative laparoscopy to check the inclusion and exclusion criteria. The following assessments should be performed: Performance Status, upper intestinal endoscopy, endoscopic ultrasound, Thoraco-Abdomino-Pelvic CT scan, PET Scan (optional), laboratory exams: serum CEA, CA19.9 and CA72.4 (optional); hemoglobin, leukocytes, neutrophils, platelets, glycemia, AST, ALT, LDH, total bilirubin, alkalin phosphatase, serum albumin, total protein, plasmatic APTT, PT and INR; creatinine clearance and serum creatinine. Staging videolaparoscopy of the abdominal cavity and peritoneal cytology testing will be carried out following the same procedure as during gastrectomy. Patients with macroscopic peritoneal carcinomatosis not visible during the preoperative morphological examinations will be excluded from the study.

### Preoperative work-up

Patients should be re-evaluated within 21 days before surgical procedure. The following assessments should be performed: Performance Status, Thoraco-Abdomino-Pelvic CT scan, PET Scan (optional), laboratory exams: serum CEA, CA19.9 and CA72.4 (optional); hemoglobin, leukocytes, neutrophils, platelets, glycemia, AST, ALT, LDH, total bilirubin, alkalin phosphatase, serum albumin, total protein, plasmatic APTT, PT and INR; creatinine clearance and serum creatinine, quality of life assessment (QLQ-C30 and QLQ-STO 22).

Patients with evidence of metastatic disease will be excluded from study.

### Surgical technique

At the opening of the abdomen, surgical exploration will be performed in order to compare data collected at preoperative videolaparoscopy with current operative findings including the effects coming from preoperative chemotherapy, namely the downstaging as far as T parameter is concerned, presence of serosal involvement and its extent, the absence of distant metastases and the feasibility of a D1-D2 resection with curative intent.

Peritoneal washing will then be performed: the abdomen will be irrigated with 200 ml of 0.9% NaCl solution (normal saline), left in the peritoneal cavity for 2 minutes and then all fluid will be aspirated from the four abdominopelvic quadrants [Left Upper Quadrant (LUQ) - Right Upper Quadrant (RUQ) - Left Lower Quadrant (LLQ) - Right Lower Quadrant (RLQ)]. All the collected fluid will be sent to the corresponding lab, where specimens for regular cytological analysis, cell blocks and immunohistochemistry (ancillary cytological study) will be sent to the Centre de Biologie et de Pathologie Est – Hospices Civils de Lyon.

All the patients will undergo a D1-D2 gastrectomy, carried out according to Japanese guidelines and to the European recommendations for the preservation of spleen and pancreas [42]: lymphadenectomy will consist of removal of nodal groups 2 to 9 as specified by the JGCRS; group 1 will be added when total gastrectomy is performed and group 12 in distal cancers. *Ex vivo* separation of lymph node groups in individual containers before the surgical specimen is submitted to the pathologist is recommended. At the end of the gastrectomy procedure the peritoneal washing will be repeated with the same procedure.

Following randomization, the patients treated into the experimental Arm receive intravenous 5-FU 400 mg/m^2^ + calcium levofolinate 10 mg/m^2^) as systemic chemotherapy induction for HIPEC 15 min before HIPEC started. At the end of the procedure the patients in the experimental Arm will undergo HIPEC.

### HIPEC techniques

The HIPEC can be carried out by open or closed abdomen technique. With the Open abdomen technique, after the D2 resection is complete, the inflow catheter is positioned in the gastric resection bed. Drains are placed through separate stab wounds in the abdominal flanks and positioned between the liver and undersurface of the right hemidiaphragm behind the spleen and in the pelvis. Number of drains used is left to the surgeon’s discretion and according to the specifications of the HIPEC circuit used. One of the two temperature probes will be secured near the tip of the inflow catheter and the other in the pelvis. Using a monofilament running suture, the skin edges are secured to the Thompson self retaining retractor, and a plastic sheath is incorporated into these sutures to create an open space beneath. A slit in the plastic cover is made to allow the surgeon hand access to abdomen and pelvis. During the perfusion all the anatomic structures within the abdominal cavity are uniformly exposed to heat and to chemotherapy. A roller pump forces the chemotherapy solution (oxaliplatin 250 mg/m^2^ with 2 Liters of G5%/m^2^) into the abdomen through the inflow catheter and pulls it out through the drains. A heat exchanger keeps the intraperitoneal fluid at 42°-43°C. After the intraoperative perfusion is complete (30 minutes), the abdomen is closed and the drains are left in place in the postoperative period until the discharge from the peritoneal cavity subsides. With the Closed abdomen technique, surgery is completed with anastomosis and drains positioning as in the Open technique, the abdominal wall sutured. External manual shaking of the abdomen will be carried out for optimization of chemotherapy distribution. The anastomoses will be constructed either before or after HIPEC administration.

### Follow-up

After HIPEC, patients will remain in the Intensive Care Unit as long as required (it will be remained at the perioperative team’s discretion, depending on patient’s needs and postoperative clinical progress). They will be evaluated with clinical examination daily. Each day for the first week and each third day thereafter, laboratory exams will be performed in order to assess haematological, renal and hepatic function. Locoregional toxicity and systemic toxicity will be evaluated according to the Common Terminology Criteria for Adverse Events (CTC-AE V4.0) from the National Cancer Institute.

Once an early postoperative follow-up four weeks after gastrectomy, the visits of follow-up are based on the date of the surgery every 3 months for the first 2 years following surgical procedure and twice a year for the last 3 years. For all patients clinical follow-up with all events and endpoints will be collected and analyzed during 5 years from their inclusion (except for patients died, lost of follow-up or expressed their refusal).

### Criteria for premature discontinuation of the patient’s study participation

Patients can be withdrawn from the study under the following circumstances: death, disease progression, initiation of alternate anti-neoplastic therapy, toxicity, intercurrent illness, non compliance (including loss of patient to follow-up), voluntary withdrawal, failure to meet the eligibility criteria.

### Premature closure of the study

Study participation by individual sites or the entire study may be prematurely terminated, if in the opinion of the sponsor, there is sufficient reasonable cause. Any investigator who wants to discontinue his/her participation to the study must immediately inform the sponsor in writing of this decision. Written notification documenting the reason for study termination will be provided to the investigator by the terminating party.

Examples of circumstances that may warrant termination include: failure to enter patients at an acceptable rate, insufficient adherence to protocol requirements, insufficient complete and/or evaluable data, frequency and/or unexpected severity of the toxicity, unacceptable toxicity. Each fatal event occurring within the 60th postoperative days will be immediately reported to the sponsor and to Data and Safety Monitoring Board (DSMB) members. The DSMB will be provided with the description of the fatal events to determine if the death has to be considered as a toxic one.

The statistical stopping rules are the following: for each toxic fatal event in the HIPEC arm, an analysis comparing the total number of patients included to the number of patients satisfying maximum toxic fatal event criteria will be performed to determine if the study should be stopped. This analysis will be realized with the Kramar sequential method [43] as implemented in the R-package [44]. This analysis will be performed using the following parameters: the maximum acceptable percentage of toxic deaths is fixed at 10% [the global alpha at 10%, and gamma at 4 (i.e., high alpha level at the beginning of the trial)], considering that the first toxic fatal event will not be taken into account.

### Sample size calculation and statistical considerations

This study is designed as a randomized phase III in order to compare the 5-year overall survival in patients with advanced gastric carcinoma randomized to hyperthermic intraperitoneal chemotherapy administration versus that of patients randomized to control arm.

The hypotheses are the following: 5-year overall survival in the control group of about 30% [9,10]; 5-year overall survival in the intervention group of about 45% (i.e. an HR of 0.67) [45]; a maximum follow-up time of 5 years; an alpha of 5%, two-sided. With this hypothesis and to have a 80% power, a total of 306 patients will have to be randomized. Since 5% of patients are expected to excluded during surgery (peritoneal carcinomatosis), a total of 322 patients will be included.

The effectiveness analysis will be carried out on the intention-to-treat population, defined as all patients included. A second analysis will be conducted on the “treated” population, determined according to the treatment actually administered (*per protocol* analysis). Overall survival will be measured from the date of surgery up to the date of death, regardless of the cause, or to the end of follow-up (5 years). The Kaplan-Meier method will be used to estimate the survival curves. Median survival will also be given and differences between the two groups assessed using a logrank test. This analysis will then be confirmed using a Cox regression analysis taking into account the center effect (frailty Cox model). The conditions for using the Cox regression analysis will be checked.

An intermediate analysis of toxicity will be realized at least every six months or every 50 patients randomized from the start of patients’ recruitment. The number of SAEs and AEs of severe (grade III-IV) with their accountability will be described.

### Ancillary study

A cytological ancillary study will be performed. The aim of this exploratory study is to compare the incidence of positive peritoneal cytology pre- and post-gastrectomy in the two arms of the study (Am A and Arm B), and to assess its impact on 5-year survival. A standardized procedure for peroperative peritoneal cytological sampling was previously approved under a large, French, multi-center prospective study (EVOCAPE 2, PHRC 2001 Funding) and will be followed for the pre- and post-gastrectomy sampling [46]. Samples will be separated into 2 aliquots. After sedimentation overnight at room temperature, the material will be centrifuged at 1500 RPM for 5–10 minutes. The cell pellet will be aspirated, smeared on Superfrost PLUS glass slide and dessicated (2 slides) or fixed with methanol (one additional). Smears will be stained with MGG in an Hematek 2000 (Bayer^©^) automaton, and with Papanicolaou. For every case, the remaining cell pellet will be submitted to the cell block technique (Shandon Cytoblock Kit®) for Haematoxylin and eosin control and immunohistochemistry. Immunohistochemistry performed on cell blocks using at least CEA (monoclonal and polyclonal) and cytokeratin 20, the Cytokine-like High Mobility Group Box 1 (HMGB1) [47] will also be considered. Positive samples will be defined by the presence of either tridimensional clusters of malignant cells, or by the presence of unequivocal isolated malignant cells. The presence of cell and nuclear atypias (cell balls not mesothelial in origin, epithelial cells with increased N/C ratio, enlarged nuclei, irregular nuclear borders, and prominent nucleoli) will be considered necessary for the positive morphological diagnosis, as previously reported [46]. Additionally, cases with CEA- and/or CK20-reactive epithelial cells will be considered positive, whatever their morphological aspect. Negative samples will be defined by the absence of malignant cells after conventional stains and immunohistochemistry (no CEA and/or CK20 immunoreactivity). Peritoneal samples taken before and after surgery will be fixed with 1/3 carbowax (20% polyethylene-glycol 1500 in 50% ethanol) and sent as rapidly as possible to the laboratory in 100 to 500 mL vials, according to the amount of fluid. Cells will be allowed to sediment overnight at room temperature. The lower part of the fluid will then be aspirated and treated as previously described (see the lab. technique) for smearing, cell blocks and immunohistochemistry.

The number of pre- and post-gastrectomy positive cytologies will be calculated. The percentage of pre- and post-gastrectomy positive cytologies will be compared using Mcnemar’s test. A Cox regression model will be used to assess the impact of peritoneal cytology on 5-year overall survival. The conditions for using Mcnemar’s test and the Cox regression analysis will be checked.

### Ethical considerations, information giving and written informed consent

The study protocol was approved by the Institutional Review Board, the Sud Est IV ethics committee on the 12 of February 2013 and the French National Agency for Medicines and Health (ANSM) on the 26 of April 2013 under the number 2012-005748-12. The study has been registered on the ClinicalTrial.gov website under the identification number NCT01882933. The GASTRICHIP study complies with the Declaration of Helsinki rules, the principles of Good Clincal Pratice guidelines and the Data Protection Act. The trial will also be carried out in keeping with local legal and regulatory requirements.

For each patient recruited into the study, written informed consent is essential prior to inclusion into the study after extensive information about the intent of the study, the study regimen, potential associated risks and side effects as well as potential alternative therapies. The investigator will not undertake any diagnostic measures specifically required for the clinical trial until valid consent has been obtained.

## Discussion

The benefit of using HIPEC as an adjuvant treatment for advanced gastric cancer has been reported in several randomized studies and two meta-analyses [38,39]. However these studies included patients who were almost exclusively of Asian origin. It has been formally demonstrated that Asian and Caucasian gastric cancers differ in terms of epidemiology, diagnosis, treatment and prognosis. On the subject of lymph node dissection for example, several randomized studies in Asia and Japan have validated D2 dissection. In Europe, two randomized studies were carried out to assess the benefit of D2 dissection and the conclusions of these studies differ from the Asian studies [8,40]. This is the reason why the benefit of adjuvant HIPEC should be evaluated on Caucasian gastric cancers.

The chemotherapeutic agents to be used for HIPEC are selected on the basis of their ability to rapidly produce a direct cytotoxic effect (their action should not be dependent on the cell cycle). The concomitant administration of heat increases the cytotoxic effect. The duration of exposure is related to the effectiveness of the intraperitoneal chemotherapy and studies investigating the pharmacokinetics of HIPEC have shown that most of the medication is absorbed during the first hour of perfusion. Furthermore, it has been shown that the rate of postoperative complication is directly linked to the duration of surgical procedures: HIPEC should therefore be administered over the shortest possible period of time in which it is able to produce its cytotoxic effect.

Mitomycin C and cisplatin are the two antimitotic agents most commonly used for HIPEC and have therefore been studied the most and shown to meet requirements for this treatment. They have been used in combination by several authors [36,48-50] for the integrated treatment of peritoneal carcinomatosis. The reinforcement of their cytotoxicity through the concomitant administration of heat and their synergistic action have both been demonstrated. Furthermore, they only generate minimal chemical peritonitis.

Good results for the treatment of gastric cancer in terms of both response and tolerance have been obtained using oxaliplatin in systemic perioperative chemotherapy protocols [51]. Recently, excellent results in treating colorectal peritoneal carcinomatosis have been reported for the use of HIPEC with oxaliplatin, with an exposure time of just 30 minutes. Furthermore, a French randomized, phase III study is currently assessing the use of HIPEC with oxaliplatin associated with complete cytoreductive surgery (CCS) to treat colorectal peritoneal carcinomatosis. These three studies used a dose of 460 mg/m^2^ of oxaliplatin for the open-abdomen technique and 350 mg/m^2^ for the closed-abdomen technique, administered in 2 liters/m^2^ of 5% glucose with the systemic administration of induction chemotherapy using FU/Leucovorin (400 mg/m^2^ 5-FU and 20 mg/m^2^ of folic acid) to increase the cytotoxic effect of the oxaliplatin. Glehen et al. recently published a report on the French experience in the curative treatment of gastric peritoneal carcinomatosis [30]. The mortality rate was high, and reached 6%. This may be explained by the fact that the gastrectomy-HIPEC association increases the risk of severe postoperative complications. It would seem preferable to arbitrarily reduce the dose of oxaliplatin (250 mg/m^2^) in order to avoid an excessive rate of postoperative complications resulting from the experimental treatment.

The incidence of signet ring cell adenocarcinoma is steadily increasing in Western countries [52,53]. Recent data has suggested not only that is has a negative prognostic value [22], but that it is also chemo-resistant [54,55] which calls into question the use of systemic neoadjuvant chemotherapy for this histologic type. Surgery remains the undisputed lynchpin of the curative treatment of gastric adenocarcinoma (subtotal or total gastrectomy with D2 lymph node dissection without splenectomy and pancreatectomy), but if systemic perioperative chemotherapy is to be recommended in Europe, the choice of perioperative treatment should be discussed on a case-by-case basis at Multi-Disciplinary Team meetings in order to take into account the patient’s pre and post-surgery general and nutritional state of health, the histology, and the preoperative and pathologic staging. Free intraperitoneal cancer cells originating from the primary tumor which invade the serosa prior to resection contribute to the peritoneal recurrence of resectable stomach cancer. Free cancer cells in the peritoneal cavity may also originate from the blood or the lymph which transport tumor cells to the surgical site. These cells may also originate from lymph node metastatis. Indeed, the surgical trauma resulting from the excision of the primary tumor provokes the release of tumor emboli in the peritoneal cavity which rapidly adhere the surface revealed by the removal of the tumor. Immediately after the intervention the resection site and traumatized peritoneal surfaces become covered in a fibrinous exudate which entraps tumor cells and protects them from the body’s defense mechanisms. This whole process constitutes the theory of what Paul Sugarbaker has called “tumor cell entrapment” [56]. This is a key phenomenon which needs to be understood in order to fully comprehend the pathogenesis of recurrence, both at the site of resection and on the peritoneal surfaces, and to assess the beneficial effects of adjuvant perioperative intraperitoneal chemotherapy.

The increased incidence of postoperative positive peritoneal cytology has only been suggested in small Asian studies [21,57]. Whilst the prognostic value of positive peritoneal cytology prior to resection seems to be well established [20,58], the same cannot be said for post-resection peritoneal cytology whose prognostic value remains controversial but which may represent a key prognostic factor for peritoneal and locoregional recurrence.

## Competing interests

The authors declare that they have no competing interests.

## Authors’ contributions

OG and LV have been involved in drafting the manuscript; SB as the methodological advisor; AG, OG, EP, GB, DV and GP have been involved in the study conception and design, assisted in writing the manuscript and have given final approval of the version to be published. All authors read and approved the final manuscript.

## Authors’ information

OG is the study coordinator, obtained the grant and is responsible for the present paper and EP is responsible for the ancillary study.

## Pre-publication history

The pre-publication history for this paper can be accessed here:

http://www.biomedcentral.com/1471-2407/14/183/prepub
